# Supplementary material for: Are carbon emissions trading and green financial instruments synergistic? -Comprehensive quantitative research based on content analysis
Source: PLoS One. 2024 Mar 7;19(3):e0298601. doi: 10.1371/journal.pone.0298601 (PMC10919601; doi:10.1371/journal.pone.0298601)
Supplement: S1 Data — (ZIP) [file pone.0298601.s001.zip › Data/地级市Z_F工作报告.zip!/║┌┴·╜¡/║╫╕┌╩╨/└┤╫╘╣1⁄2╓┌║┼í╛┬φ┐╦╩2╛▌═°í┐_2008.docx]

各位代表：

　　我代表鹤岗市人民政府向大会作政府工作报告，请予审议，并请各位委员提出意见和建议。

　　一、2007年工作回顾

　　过去一年，我们在省委、省政府和市委的正确领导下，以科学发展观为指导，坚持以经济建设为中心，以改善民生为根本目标，克服了上年财政一次性收入比重大、经济增长空间小、项目建设门槛提高、农业遭受特大旱灾等困难，圆满超额完成了上次人代会确定的目标任务，开创了全市又好又快发展的新局面。

　　——主要经济指标快速增长。在连续几年经济较快增长的基础上保持了较快的增长速度。预计地区生产总值完成156亿元，比上年增长13.2%，三次产业分别增长2.6％、20.9％、11.6％；财政收入完成17.8亿元，增长10.6％，按可比口径增长20.8％；固定资产投资完成55亿元，增长28%；社会消费品零售总额增长15.6%，创近年最好水平；城镇居民人均可支配收入和农民人均纯收入增长8％和10.3％。规模以上工业实现增加值42亿元，增长22％，实现利税12亿元，增长16.5%。进出口总额实现4100万美元，增长51.5％。金融机构存贷款余额达到175亿元和130.25亿元，比年初增加13.26亿元和8.8亿元。

　　——三年“项目建设年”取得重大成效。以大项目建设为标志，“项目建设年”决战决胜年任务圆满完成。全年实施工业项目40项，完成投资9亿元，三年“项目建设年”取得重大成效。大项目建设成效显著，全市瞩目的120万吨甲醇项目通过国家环评，10万千瓦矸石热电项目得到国家批复，百万吨化肥在国家待批；电厂二期、30万吨玉米深加工等项目投产运行，70万吨玉米加工、日处理600吨鲜奶等项目加快推进建设。中小项目形成规模，木糖、米糠油加工、康维生物、矿山配套机械加工等一批高新技术、非煤产业项目建成投产，稻壳供热发电、秸秆发电等项目进展顺利，洗精煤规模扩大，效益大幅提高。老企业改造项目有新进展，华隆玻璃、东华水泥、工业布厂等老企业焕发新的生机。园区建设力度加大，投入4600万元用于土地储备和基础设施建设，园区承载项目功能更趋完善。

　　——新农村建设扎实推进。农业发展经受住严峻考验。动员全市力量开展抗灾自救，战胜了百年不遇特大旱灾，成为历史上第二个丰收年。采取有效措施减灾增收，确保了大灾之年少减产不减收。粮食总产实现11.3亿斤，农业增加值实现12.8亿元，增长14％；畜牧业迎来难得的市场机遇，生猪、奶牛、肉牛饲养量分别增长7.1％、7.1％和24％，畜牧业增加值增长8.2％。产业化步伐加快。省级龙头企业达到8户，三级龙头企业带动3.3万户农户户均增收900元。场县共建实现新突破。完成跨区作业124.5万亩，推广垦区先进适用技术100万亩，垦地医疗合作在全省介绍经验。农村面貌有新改观。加大投入力度，加强了农村公路、饮水工程等项目建设，改造农村土草房1860户，进一步解决了农民最企盼、最渴望解决的“供水、行路、住房”等重点问题，农村面貌进一步改观。

　　——对外开放水平进一步提高。成功举办“俄罗斯·中国年”活动；锰矿开采、石油天然气开采等对俄合作项目运作顺利，水泥生产、房地产开发、砖厂建设、油轮租赁等项目达成合作意向；口岸过货18.21万吨，增长18.4%，过客2.86万人次。华祥粮油成为我市第一家跨国贸易企业，实现了跨国经营和自主品牌出口。利用外资创历史最好水平，外资到位614.5万美元，增长2倍。招商引资成效显著，市场招商完成46.23亿元。 

　　——社会事业全面进步。科技创新提档升级。获得国家和省级科研成果2项，省级高新技术企业达到16户；企业与大专院校、国际科企实现科技项目合作7项；信息化建设稳步推进，实施了8个企业信息化示范工程和2个农业信息化试点。教育事业快速发展。办学条件进一步改善，学校基础设施建设继续加强；教学质量稳步提高，普通高考再创佳绩，师专迎评顺利通过。卫生体系逐步完善。公共卫生、农村卫生和社区卫生体系建设得到强化，农村卫生基础设施建设步伐加快，公共卫生突发事件应急处理长效机制逐步健全，重大疾病预防控制进一步加强。文化创作取得历史性成果。我市创作的相声《免费电话》实现打入央视春晚零的突破，文艺赛事获省级以上奖励42项，群众性文化活动更加活跃。体育事业发展加快。体育设施进一步完善，群众性体育活动全面开展，开工建设市综合训练馆，成功举办了市八运会。劳动和社会保障不断强化。实现就业再就业27241人次，开发公益性岗位975个，发放小额担保贷款810万元，城镇登记失业率控制在3.9%以内。环境保护继续加强。全市工业节能8万吨标煤；哈啤公司废水处理、鹤选总厂洗煤废水处理和兴安矿矿井水净化利用工程顺利竣工，化学需氧量削减2277吨，生态建设项目为近年最多。民族、宗教、外事、侨务、审计、监察、双拥、国防、人口与计生、人防、档案、司法、物价、统计、地震、残联、老龄等各项事业都取得新成绩。

　　——安全稳定明显加强。安全形势稳定好转。以制度建设为保障，严格领导责任和安全监管，安全形势稳定好转。全市各类安全事故死亡人数下降29％，比省控制指标下降31.6％。加强煤矿安全专项督察，地方煤矿和鹤矿集团都杜绝了三人以上重特大事故；非煤工矿商贸安全形势稳定，交通事故明显下降，消防安全连续12年无重特大火灾，安全生产综合水平位居全省第二位，实现了市矿双平安。社会治安形势稳定。加强公安基层基础建设，完成了两县指挥中心“三台合一”； 积极推进“平安鹤岗”建设，严厉打击各种刑事犯罪，刑事案件发案率下降，现行命案破案率达93.6％。信访工作得到加强。畅通群众诉求渠道，积极处理信访积案，及时排查矛盾，依法维持信访秩序，信访案件总量大幅下降，进京到省集体访下降26％、8.4％，个体访下降39.6％、94％。

　　——促发展、保民生、利长远的51件大事实事全部落实。市政府及有关部门加大力度、创造条件、强化管理，51件城乡基础设施建设、社会事业项目和直接惠民利民的大事实事按计划完成，其中筹资11亿元，全部完成46件。城乡基础设施建设继续加强。哈肇公路名山至肇兴段、新华工业园区道路拓宽改造工程竣工通车，建设330公里农村公路，硬化村内街巷路44.4公里；国土综合治理、矿山环境治理项目全部完成。城市建设再上新水平。11条城市主次干道、62条街巷道路、宇南桥建成投入使用；大规模开展城市绿化，改造了五指山公园、新世纪广场；城区“一河四沟”防洪治理任务全部完成；供热三期新增供热面积200万平方米，城市供水管网改造新增供水用户4500户，完成68栋楼房陈旧供热设施改造；街巷路、居民区亮化继续加强，新增路灯422基，亮灯率达到98％；城市小区的内部配套设施建设得到完善，100座公厕建设任务全部完成；棚户区改造工程开始启动。社会事业发展项目全部完成。6000平方米学校危房改造、师专图书馆宿舍扩建完成，职教中心、卫生中专、鹤矿集团实训基地、市一中1万平方米教学楼和4000平方米图书馆建成投入使用。改造建设了5所乡镇卫生院，启动了萝北县新型农村合作医疗，全市参合率达90.3％；社区卫生服务人口覆盖率达80％，标准化率达80％；兴山矿医院主体工程完工；三个农村客运站投入使用，防汛抗旱指挥中心建成。开工建设了20处农村饮水工程，解决了1.1万人的饮水安全问题。新建改造了10个基层派出所，增加了公安消防特种设备。建成无线电视转播台，实现了中央第7套无线电视和中央1套调频广播、省交通广播落地播出；增设51套全民健身路径及公共体育设施。强化基层基础管理，全部解决了社区办公用房。人民群众得到更多实惠。机关事业单位工作人员调资和公务员津贴补贴全部兑现，财政供养人员的热费补助发放到位。将10100名破产、关闭、改组、改制企业的退休人员纳入基本医疗保险。加强了解困救助，提高了低保补助标准，增发1200万元低保金；社会福利家园、老年活动中心投入使用；沉陷区治理二期工程完成楼房建设62万平方米，安排住户6402户，建设了5000平方米廉租房；建立了大病救助机制；发放政府专项救助资金525万元；全部免除两县农村中小学杂费，免除市郊农村5976名贫困学生杂费，为13756名学生提供免费教科书，救助了4000名贫困学生。

　　5件跨年度的大事实事正按计划实施。环山、鹤南和东郊电网工程建设进展顺利；小鹤立河水库扩建工程完成前期工作；水源保护区综合治理工程通过环保评审；城市污水处理厂建设工程获得国家批复；社区干部全部纳入养老保险体系待社区换届后即可实施。

　　比年初计划新增投资4760万元，完成了萝北团结镇土地整理、市区校园基础设施维修、有线电视光缆铺设和数字传输设备改造、12万平方米城市小区硬化绿化、30条道路排水等五项城乡建设、社会事业发展项目。上年结转的千米演播厅、4000平方米老年活动中心投入使用。

　　——政府自身建设不断加强。推进行政审批制度改革，减少40项审批事项；健全了突发社会安全事件、重特大事故、突发公共事件等应急处理机制。建立了人才激励机制，出资110万元对优秀拔尖人才进行了奖励。充分发挥人民办事中心职能作用，受理各类业务11.5万件，办结率98％，群众满意度达95％以上。 

　　过去的一年，是我市经济持续快速发展的一年，各项社会事业、重大基础设施建设全面进步的一年，人民群众得到更多实惠、生活水平显著提高的一年，政治社会更加平安稳定的一年，和谐社会建设取得重要进展的一年。这是省委、省政府和市委正确领导的结果，是市人大、政协依法监督、民主监督的结果，是全党抓经济、全民创业、全社会招商、全区域上项目、合力攻坚的结果，是全市各级组织、广大干部群众共同奋斗的结果，是中省直部门和社会各界大力支持的结果。在此，我代表市政府向全市人民和各级干部，向历届老领导、老干部，向驻鹤部队、武警官兵和公安司法干警，向关心、支持和贡献鹤岗发展的市内外投资者、社会各界人士和市人大、政协、各民主党派、人民团体表示衷心的感谢和崇高的敬意！

　　在取得重大成绩的同时还存在一些不可忽视的问题，主要是经济总量小，发展速度慢，产业层次低，产业结构不合理，城市转型难度大；由于国家宏观调控力度加大和松花江十年休养生息，项目建设条件从严、门槛提高、难度加大；新的经济增长点尚未有效形成，新上项目投产达效还需要一定时间，加之鹤矿集团继续限产，小煤矿继续关闭，财政增收难度加大；社会事业发展滞后，社会矛盾仍然突出，治安、信访、安全生产等任务依然艰巨；抓经济发展的氛围、合力、环境还有很大差距，急需加以改善。

　　二、2008年工作安排

　　今年是深入贯彻十七大精神、全面落实科学发展观的重要一年，是我市发展史上意义重大的关键一年。全市工作总的要求是：坚持以十七大精神为统领，全面贯彻落实科学发展观，实事求是，与时俱进，遵循经济社会发展规律，从鹤岗实际出发，坚持和完善已确定的正确的发展战略、思路和措施，解放思想、改革创新、强力推进、合力攻坚，发挥后发优势，实现捷径赶超，努力推动经济社会又好又快、更好更快发展和各方面工作全面进步升级，全面发展社会事业，改善民生，建设和谐社会，打好经济社会持续快速发展的基础，加强政府自身建设，为城市创新转型和人民富裕幸福做出更大贡献。

　　主要预期目标是：地区生产总值增长12%，财政收入增长10%，固定资产投资增长25%，社会消费品零售总额增长15%，城镇居民人均可支配收入、农民人均纯收入分别增长10％、7％，居民消费价格指数涨幅控制在5%左右。

　　（一）强力推进项目建设，加快工业经济发展

　　实施工业立市战略，转变发展方式，优化经济结构，走新型工业化道路，务求又好又快、更好更快发展。规模以上工业增加值增长15％，利税增长12％。

　　开展新一轮三年“项目建设年”活动。继续举全市之力锲而不舍、一以贯之抓项目，开展第二轮为期三年的“项目建设年”活动，使主导产业重大项目、资源精深加工项目、高新技术项目、重大基础设施项目、大企业大集团项目、民营经济项目、县区项目、区域内各大经济单元项目、领导干部带头招商和社会招商项目等十个方面项目建设取得突破性成效。今年要实施重点工业项目40项。全力抓好120万吨甲醇、200万吨水泥、中医药大学产业园、禾友农药异地改造、秸秆发电等18个新项目开工建设和70万吨玉米深加工、10万千瓦矸石电厂、日处理600吨鲜奶等22个续建项目建设，确保矿山配件、巨源碳素异地改造等12个项目竣工投产，力争100万吨化肥、120万千瓦电厂三期、鸟山煤矿、绥滨松花江大桥等项目获得批复，积极做好关门嘴子水库、鹤名铁路、煤层气开发、中海油120万吨磷肥、龙煤集团120万吨甲醇、5000吨木糖、腐植酸基地、萝北石墨深加工和华欣纸业启动、180万千瓦龙江电站等项目的前期、审批和招商。搞好老企业技术改造项目，抓好大豆、猪肉、水稻等精深加工。

　　大力发展循环经济。转变工业发展方式，提高资源能源利用率，实现变废为宝。建设秸秆发电、5000吨木糖扩产、米糠油二期、保健调味品、稻壳发电等项目，扩大农副产品循环利用规模。建设煤层气、煤矸石、粉煤灰综合利用、矿井水净化工程和再生纸项目，提高废弃物综合利用水平。开发风能、水能、太阳能综合利用项目。

　　加快工业园区建设。完善园区规划设计，投入1000万元加强基础设施建设。出台《工业园区优惠政策和管理办法》，组建园区管委会，加快企业入驻。

　　加强工业运行服务调控。强化服务调控，及时预测分析工业运行形势，协调煤、水、电、运、资金等生产要素，全力支持重点企业和投产项目，提高经济效益，争取新增规模企业10户。

　　（二）全面发展农村经济，扎实推进新农村建设

　　农业增加值增长6％，畜牧业增加值增长10％。

　　加快绿色特色农业发展。增加有机、绿色、无公害食品种植面积。坚持标准化生产，立足抗灾夺丰收。推进“主辅换位”，加快畜牧业发展。抢抓市场机遇，扩大规模经营，提高集约化、科技化、专业化养殖水平。发展农民专业合作组织，加快土地经营权流转，扩大农业生产经营规模。

　　推进产业化经营。扶强扶壮龙头企业，延长产业链条，实施精深加工。围绕龙头企业需求，建设生猪、奶牛和玉米、水稻、大豆专业化、规模化、优质化、标准化生产基地，引导龙头企业和农户建立风险共担、互利双赢的利益联结机制。

　　加强农业基础建设。加快水库扩容改造、除险加固工程建设，提高供水能力。推进江萝、德龙灌区建设。完成塘坝、灌区田间配套工程、涝区清淤等整治工程，增强抗灾能力。

　　加快农业科技推广。实施科技园区行动计划，将各项农业适用新技术综合组装配套，农机农艺、良种良法有机结合，提高农业标准化水平。加强动植物疫病防控，加强农产品质量安全。

　　扎实推进新农村建设。巩固试点村成果，注重面上推进。探索建立集体资产长效管理机制，强化对已建成项目的管理和维护。继续建设通乡村农村公路，建设饮水工程，解决农村饮水安全问题。全面发展农村社会事业，完善合作医疗、农村低保、五保户供养等社保救助体系。

　　（三）加强基础设施建设，改善人居和发展环境

　　适应大项目建设加快、经济总量持续跃升、民生需求增加的需要，加快重点基础设施、配套设施建设。

　　突出规划的龙头地位。实施好第三轮城市总体规划，做好城市风貌特色、控详和专项规划；坚持城乡统筹，编制两个县域体系、50个村屯建设规划。以规划为引领，全面推进城市建设的节能、节地和环保。

　　加强基础设施建设。启动建设棚户区改造工程。加快道路、供电、供热、防洪、河道治理和污水、垃圾、医疗废物处理等基础设施建设，完善城市配套设施，加快住宅建设，改善人居环境，增强承载能力。大规模搞好城市绿化，加快建设生态园林城市。按照“城在林中、楼在树中、人在绿中”的目标，实施“点、线、圈、面”全方位绿化，扩大城市森林覆盖率。

　　创新城市管理方式。加强城市管理，实施卫生、市容、交通、市场、河道、物业等重点专项整治活动，努力提高市民文明素质。健全管理长效机制,及时解决城市管理服务诉求，推动城市管理人性化、科学化、法制化。

　　今年要投入10.74亿元完成15件城市建设大事实事。

　　1. 投资4.1亿元开工建设15万户居民煤层气燃气利用工程；

　　2.投资2.4亿元开工建设沉陷区治理二期20万平方米住宅工程；

　　3.投资2.04亿元建设日处理５万吨城市污水处理工程； 4.投资9700万元开工建设生活垃圾无害化处理厂；

　　5.投资1500万元建设日处理５吨医疗废物处置中心；

　　6.投资6000万元完成供热三期工程，新增供热面积100万平方米；

　　7.投入1000万元改造100栋居民楼陈旧供热设施，实施分户供热；

　　8.投资1500万元修建10条城市主次干道；

　　9.投资300万元硬化60条街巷路；

　　10.投入700万元建设麓林山自然公园，改造天水湖公园，规划建设北山自然公园；

　　11.投入500万元完成城市规模绿化；

　　12.投入290万元建设100座公厕；

　　13.投入200万元亮化12条街巷道路；

　　14.投入220万元建设小鹤立河防洪整治工程；

　　15.投入120万元增设500个果皮箱、200个垃圾箱。

　　要投入5.55亿元实施17件城乡基础设施建设大事实事。

　　1.投入1.2亿元完成环山、鹤南和东郊等区域电网建设；2.投入1.83亿元完成小鹤立河水库扩容5200万立方米；3.投入9000万元建设300公里农村公路；

　　4.做好82.3公里萝北至嘉荫公路前期工作，力争开工建设；

　　5.投入2439万元实施土地治理；

　　6.投入1080万元建设两座塘坝，新打100眼补水井；

　　7.投入1000万元实施矿山治理；

　　8.筹资2000万元进行沉陷区土地整治；

　　9.投入1000万元开工建设占地８万平方米公路货运物流中心；

　　10.投入500万元治理33平方公里水土流失；

　　11.投入900万元完成元宝山水库除险加固；

　　12.投入400万元建设18处农村饮水安全工程，解决１万人的饮水安全问题；

　　13.投入200万元对城区河段进行防洪治理；

　　14.投入100万元建设１处国家级旱田节水示范区；

　　15.投入90万元新建三个农村客运站；

　　16.投入5500万元实施小鹤立河、细鳞河、五号水库上游污染治理；

　　17.投入1000万元启动总投资5亿元的绥滨松花江大桥前期工作。

　　（四）扩大对内合作、对外开放，提高经济开放合作水平

　　顺应国际经济分工合作加速调整、国内省内功能区划分加快实施的潮流，拓展合作领域，提升开放层次，打造开放型经济新优势。完成进出口额4920万美元，利用外资737万美元，招商引资70亿元，分别增长20％。

　　提升对外经贸层次。加快推进赴俄锰矿开发、石油天然气、水泥厂、房地产开发、砖厂项目。扶持鑫诚经贸、萝北边贸两户企业发展对俄商业包裹贸易，支持华祥粮油、宝泉岭扩大对韩豆制品出口规模，推进对俄出口水泥、玻璃瓶、家电、木制品等项目，扩大外贸规模。积极推进台湾润泰集团“大福源”超市等项目，提升利用外资水平。优化口岸环境，加快名山第二港区建设，新建口岸货物仓库。

　　加大招商引资力度。学习借鉴先进经验，总结自身实践，探索工作规律，加大工作力度，完善推进机制，提高招商引资成效。要突出招商重点，主攻项目、资金、技术、人才和经济实力强的先进发达地区、同类产业聚集区、国内外大企业、大集团、大专院校、科研院所，务求有大的突破。进一步创造宽松优良的软环境，规范统一优惠政策，完善考核办法，提高工作成效。

　　深化区域合作。搞好区域共建。整合域内资源，统一发展战略，统一向上争取，统一优惠政策，统一协调服务，帮助各大经济单元更多地争取上级支持，使其成为各自领域内最好最快发展的重点单位，实现区域经济集约集群发展。发挥鹤矿集团资源实力优势，做深做大煤炭加工业，积极发展“吃配”经济；发挥宝局资源、技术装备、经济优势，发展优质绿色食品加工业，扩大场县合作共建，促进农业现代化、规模化和产业化；发挥华能集团行业优势，合力运作电厂三期工程，做大煤电产业；发挥鹤北局林木资源优势，引进战略合作伙伴，做强林木加工业。大力发展县域经济。落实优惠政策，加速推进县域经济工业化、产业化、城镇化和现代化，加快项目建设、基础设施建设和社会事业发展，增强县域经济实力。加快发展城区经济。推进体制机制创新，强化完善区级功能，在经济发展、改善民生、城市建设管理、公共服务中发挥更多更大的作用。

　　（五）加快发展民营经济和第三产业，培育新的经济增长点

　　打造全民创业强势。要加强政策扶持，鼓励民营企业调整产业结构，投资新兴主导产业，参加社会事业和基础设施建设。引导有规模的企业按市场经济规律加强内外联合，组团投资创业、做强做大，建立现代企业制度，强化管理，提高效益。实行内资外资同等优惠政策，调动保护民营企业家在家乡创业发展的积极性。

　　加快发展第三产业。发展现代流通业。积极推进连锁经营、配送经营，深入实施“万村千乡”工程，提高“农家店”覆盖率和配送率，引进大集团，打造新型商业业态。完善现代物流体系，建设公路货运物流中心，筹建万圃源农产品物流中心。发展现代服务业。大力发展金融保险、中介服务、信息咨询等行业，支持发展特许经营、代理制等现代经营方式和组织形式，加快发展社区、养老、物业管理等服务业。

　　做大做强旅游业。以创建全国优秀旅游城市为契机，发挥小兴安岭与三江平原交汇、黑松两江之间特殊地理位置所独有的山、水、边境特色风光游资源，深入挖掘提升小兴安岭森林、黑龙江界江、矿山开发建设的旅游功能以及抗联文化、知青文化、垦荒文化、少数民族风情文化、边境异国文化的内涵，增强软实力、吸引力、竞争力。突出重点，加快基础设施和旅游景点景区建设，与嘉荫联合开通黑龙江界江游航线，建设黑龙江自然史馆，开发建设名山特色游乐园。加强宣传推介，把“小兴安岭——黑龙江山、水、界江避暑乘凉风光游”打造成“南海北江”国内外知名旅游黄金线，建设黑龙江东北部旅游胜地。

　　（六）做好财税金融工作，增强经济社会发展支撑力

　　加大财政金融对经济的支持，努力培植财源。抢抓全市快速发展的机遇，增强财政金融筹资能力，增强对经济发展、社会事业和民生保障项目的支持力度。加大财税征收力度，坚持依法征税、应收尽收，确保财税收入稳定增长。

　　围绕经济社会发展重点，调整财政支出结构。控制一般性支出，坚持公用财力向经济社会发展、基础设施建设、公共服务、惠民助困上倾斜，保证干部群众收入增加、惠民利民项目和扶贫助困资金需求。

　　改善金融生态环境，为又好又快发展提供资金支持。加强银政、银企合作，增加对企业特别是中小企业的贷款投放。支持深化金融改革，提高企业信用等级，防范金融风险，建设金融生态区。

　　(七) 推进节能减排，加强环境保护

　　加大推进力度，逐步形成节约资源和保护生态环境的产业结构、增长方式、消费模式。万元生产总值能耗下降4.5%，化学需氧量削减1860吨以上，二氧化硫排放量在2005年基础上削减365吨。

　　积极推进节能减排。完善工作机制，建立政府引导、企业为主、社会参与的节能减排投入机制，确保节能减排措施落实到位。制定政策，引导企业加快技术改造和淘汰落后工艺、技术、装备，提高资源利用率和回收利用率。加快节能减排工程建设，搞好污染源普查，实施绥滨电厂等11项松花江治理规划项目。

　　大力实施环境整治。加快污染物排放治理和水源治理工程建设，加强矿山环境治理和水土流失治理。加强城市空气污染治理，大力推行集中供热，继续拆除小烟囱，逐步推广洁净燃料，控制汽车尾气污染，努力改善空气质量。加强退耕还林、湿地保护、防护林等工程建设。

　　（八）强化社会建设，实现统筹协调发展

　　积极争取国家和省产业政策支持，增加财政投入，加强市场运作，发动社会力量，提升城市经营，加快社会事业建设，增加公共服务，让人民更多地共享发展成果。

　　加快发展科技事业。加大科技投入，形成企业自主、政府扶持的科技创新体系；坚持“产学研”相结合，支持企业组建5户研发机构；积极引进先进科技成果，吸引更多的高等院校、科研院所把研发基地、实验基地建在鹤岗。

　　优先发展教育事业。统筹区域教育协调发展，规范办学行为，深化教育改革，促进教育公平。调整优化5所学校布局。加快普及高中阶段教育，大力发展职业教育，加强高等教育内涵建设，支持发展民办教育、社区教育。

　　大力发展卫生事业。强化公共卫生、农村卫生和社区卫生体系建设，建立覆盖城乡的基本卫生保健和医疗救助制度，为特困群众提供免费公共卫生服务和低收费的基本医疗服务。完善公共卫生突发事件应急处理长效机制，健全重大疾病预防控制体系。

　　积极发展体育事业。增加体育设施，普及全民健身运动，增强竞技体育实力，扩大体育产业规模。

　　加强人口与计划生育工作，人口自然增长率控制在3‰以内。

　　全面加快民族、宗教、外事、侨务、双拥、国防、人防、档案、物价、统计、地震、残联、老龄、妇女儿童等各项事业发展。

　　今年要投入1亿元完成15件社会事业大事实事。

　　1.投入100万元改造教育两个幼儿园；

　　2.投入300万元完善市特教中心学生食堂和宿舍；

　　3.投入300万元改善５所学校校园场地；

　　4.投入200万元完成2000平方米学校危房改造；

　　5.投入100万元新建２所社区卫生服务机构；

　　6.农村新型合作医疗实现全覆盖；

　　7.投入1500万元兴建8500平方米体育综合训练馆；

　　8.投入460万元新增全民健身路径器材50套；

　　9.动员社会力量建设５个市级业余体育训练基地；

　　10.争取资金200万元建设标准化街道办事处、社区；

　　11.投入3800万元成立鹤岗市技师学院、就业培训中心、人力资源服务中心和公共实训基地；

　　12.规划建设市博物馆；

　　13.启动建设市文化活动中心；

　　14.投入700万元建设黑龙江自然史馆；

　　15.建设名山岛游乐园。

　　（九）大力改善民生，巩固发展社会和谐稳定

　　坚持以人为本，重在改善民生、增加民利、救助民困，下大力气解决好人民群众最直接、最现实、最根本的利益问题，维护社会公平，提高和谐社会建设水平。

　　加强就业再就业工作。认真贯彻落实《就业促进法》和《劳动合同法》，实施积极的就业政策，以创业带动就业，增加就业岗位，多渠道扩大就业。提高就业质量，增强就业稳定性，争取“零就业”家庭动态管理为零，开发公益岗位600个，实现就业再就业2.3万人次，城镇登记失业率控制在4.5％以内。整合培训资源，加大职业培训力度，提高劳动者素质。落实最低工资制度，创建和谐劳动关系。

　　完善社会保障。做好社会保险工作。加大“五险”扩面力度，强力推进高风险行业参险，稳步实施农村养老保险，扩大社会保险覆盖面，增加基金积累，提高保障水平。完善社会救助体系。管好用好政府专项救助资金，提高城乡低保标准，支持社会慈善、社会捐赠、群众互助等救助活动，加强救灾救济工作。加快社会福利设施建设。鼓励民间力量兴办社会福利事业，形成投资主体多元化、服务对象公众化、运行机制市场化、服务方式多样化、服务队伍专业化的社会福利体系。

　　全力加强安全生产。全面落实安全生产责任制，继续实行领导包保责任制、几大班子合力抓安全、安全专项督查、事故责任追究特别规定等机制制度，深入开展隐患排查治理、标准化生产活动，严肃查处违法违纪和失职渎职行为，坚决杜绝和遏制煤矿重特大事故。建立健全突发事件应急机制，强化道路交通、消防、危险化学品、食品、药品、森林防火等各项安全，有效控制各类事故发生，确保全市安全生产形势稳定好转。

　　做好信访工作。深入开展矛盾排查调处，积极预防和妥善处理群体性事件；完善信访领导责任制，强化基层接访和“谁主管谁负责”、“一级对一级负责”工作机制，健全信访稳定工作责任追究制度。

　　深入创建“平安鹤岗”。强化社会治安综合治理，严厉打击各种违法犯罪，确保人民生活安宁。加强基层组织建设。增加投入，加强社区、社会中介组织等基础基层组织建设，完善服务管理，提高全社会的组织化程度和公共管理水平。

　　加强价格监控。开展“价格诚信”活动，加强对市场的监控调控，规范价格秩序，防止物价过快增长。

　　今年要筹资2.35亿元完成直接惠民利民的16件大事实事。

　　1.筹资近亿元解决国有“并轨”企业拖欠职工工资问题； 2.筹集1200万元增发城市低保金，增加月补助10元，超出全省平均水平；

　　3.筹资150万元提高农村低保年人均补差额100元；

　　4.新建5000平方米廉租房交付使用，支付620万元资金确保6000户以上家庭享受货币补贴，实现符合廉租住房保障条件的家庭应保尽保；

　　5.投入500万元再建一座老年公寓，“五保户”集中供养率达到60％；

　　6.投入200万元建设人民信访接待大厅；

　　7.投入800万元再建两处老年活动中心；

　　8.鼓励发展民间福利事业，使各类福利机构的床位数超过全省平均水平；

　　9.投入500万元对20个村进行扶贫开发；

　　10.筹集50万元建立优抚对象大病救助资金；

　　11.在煤矿、家政服务、餐饮服务业实行最低工资标准； 12.落实结核病控制措施，提供免费医疗；

　　13.投入8000万元兑现今年新增的国家工作人员和事业单位人员津贴补贴；

　　14.扩大专项资金，对特殊弱势困难群众进行救助；

　　15.增加小额担保贷款和公益岗位，促进创业就业；

　　16.专项开展困难学生助学，保证无一人因贫失学。

　　按照国家批复，我市棚户区改造工程总投资68.9亿元，其中配套公建设施项目政府投资12.65亿元；总建筑面积477万平方米，其中住宅418万平方米，投资54亿元，安置住户6.7万户，20万人受益，将在五年内完成。按照2007年——2008年计划，将开工8个住宅区，公建配套设施投资5.5亿元。

　　今年计划投资3.15亿元开工建设12项重点工程。

　　1.开工建设3万米供水管网；

　　2.开工建设6.4万米排水管网；

　　3.开工建设5万米供热管网；

　　4.开工建设4.1万米燃气管网；

　　5.开工建设8.8万米供电线路；

　　6.开工建设44万平方米道路；

　　7.开工新建3所中学，新建维修4所小学；

　　8.开工新建维修4所医院；

　　9.开工新建4所卫生所；

　　10.开工新建6个幼儿园；

　　11.开工新建5个派出所；

　　12.开工新建61个居民委。

　　（十）繁荣社会主义文化，提高软实力、竞争力

　　文化需求是人民群众生活最基本最重要的需求，是经济发展的核心竞争力。在全面建设小康社会的今天，文化需求更显重要、紧迫和突出。要迅速掀起文化建设新高潮，努力实现大发展大繁荣，增强软实力、竞争力，为人民幸福、经济社会发展注入强大生机活力。

　　树立大文化理念。着眼于全体人民科技、文化、文明素质提高，学习共享国内外先进文化，丰富人民群众日常文化生活，加强文化设施建设和文化产业发展，办好专业文化团体，开发提升地方特色文化资源，全面提升文化软实力、核心竞争力。

　　培育优秀城市文化。大力加强地方城市文化建设，加快矿区、抗联、垦荒、知青以及黑龙江流域等独具地域特色的历史文化的挖掘、整理和开发；加强机关、企业、社区、乡村、校园、家庭文化建设，大力培育鹤岗人民的主人精神，增强主人意识，履行主人责任，规范主人行为，树立主人形象，保障主人权力，展示主人风采，热爱家乡，共同建设发展家乡，共享家乡建设发展进步成果。要重点营造和培育文明开放、干事创业、务实创新、竞争图强的城市文化，使之成为鹤岗捷径赶超、跨越发展的强大精神动力、核心竞争力。

　　 繁荣文化事业。以弘扬鹤岗精神、培育城市文化为主题，实施精品创作工程，力争获得国家级、省级奖项；活跃文艺演出，实施文企共建工程，大力组织文艺专场演出，并组织文艺宣传小分队深入学校、企业、社区、军营巡回演出；开展群众性文化活动，以“煤之韵”群众文化活动周、改革开放30周年、少儿文艺调演等活动为载体，开展全市性大型群众文化活动；以文化科技下乡、建设农家图书馆、创建农家文化大院为牵动，着力丰富农村、林区、垦区、边远地区的文化生活。

　　加快文化基础设施建设。扩大有线电视入户率，增强数字化传输能力，加快自然村广播电视“村村通”工程建设；办好鹤岗政务网、新闻网，筹建鹤岗广播电视网站。加快博物馆、图书馆、综合文化活动中心、黑龙江自然史馆等标志性文化服务设施的规划建设。

　　三、加强政府自身建设

　　我们必须清醒而敏锐地看到，在全面建设小康社会、振兴东北老工业基地的大潮中，经过三年“项目建设年”的成功运作，我市确有一批决定鹤岗经济全局和历史命运的新产业大项目在加快运作，有实质性明朗化突破，将在新一轮“项目建设年”中大规模开工建设，这在鹤岗历史上前所未有；以沉陷区、棚户区改造为载体，加之随着经济发展、实力增强、科学发展观理念升华、更加注重社会事业发展，今后三、五年我市社会事业项目将大规模开工建设，这在鹤岗历史上前所未有；与之相适应相配套的供、排、灌水等水利工程项目，电、气、路等基础配套项目，污水、垃圾、废弃矿体、山体、国土整治等环保项目和城市基础设施项目将大规模建设，这在鹤岗历史上前所未有。重大经济发展项目、社会事业项目、基础设施项目全面同步大规模建设，更是鹤岗历史上前所未有，使鹤岗进入老工业基地振兴、城市创新转型、全面建设小康社会发展建设的高潮期、攻坚期、机遇期、收获期。这是几代鹤岗人长期艰辛努力、企盼期待的大势好事，使人震撼、令人振奋。但同时也呼唤考验着全体鹤岗人民的胆识、智慧和力量，依赖期待着鹤岗人才、精英、干将、功臣、实干家、企业家脱颖而出、卓绝努力、大有作为，考验检验着各级班子、领导干部的素质、能力和作风。大规模的发展建设创造着巨大的效益、巨大的希望，也带来了巨大的工作量、巨大的困难和风险。机遇风险共存同在、相互转化，机遇稍纵即逝，搞得好，是大效益、大福祉、大贡献，反之就是损失、挫折和责任。现在是需要我们严肃回答“你们准备好了吗”这一问题的时候了，我们必须加紧准备，必须准备好，不打无准备无把握之仗，战则必胜，每战必胜。我们必须解放思想、与时俱进，横下一条心，拧成一股绳，勇敢迎接这一战略机遇期、发展建设新高潮，勇敢面对一切风险和困难，依靠上级领导的支持和全市人民的智慧力量，合力强力攻坚，推动这一重大战略机遇和发展建设高潮迅猛到来，强势发展，取得重大成效，造福鹤岗人民，贡献鹤岗历史。

　　（一）切实加强学习，努力提高领导素质。要深入学习党的十七大精神，学习中国特色社会主义理论，学习科学发展观的重大理论、深刻内涵，学习中央经济工作会议、省委市委全会精神，学习一切需要熟悉的新知识、新技术、新经验，努力提高坚持中国特色社会主义道路、落实科学发展观、推进思想解放改革开放、加快经济社会发展、构建和谐社会、造福全市人民的自觉性、主动性、责任感、紧迫感，努力做到通过理论上的高度成熟实现政治上的高度成熟，通过知识水平的明显提高实现领导素质的明显提高。

　　（二）加强实践磨练，努力提高领导能力。要适应新形势新任务的要求，努力提高领导经济工作和解决实际问题的能力。市委全会明确提出了建设“发展型、民生型、和谐型、廉洁型”班子的目标，符合我市实际，政府班子要坚决落实，抓出成效，真正把政府各级领导干部和各级班子建设成为懂经济、重民生、善于解决复杂困难问题、能打硬仗恶仗、能独当一面、能打开局面、靠得住、过得硬、有能力的优秀领导人才和坚强领导集体。

　　（三）廉政勤政，搞好作风建设。必须严格依法行政，自觉接受人大的法律监督、政协的民主监督和全市人民的公共监督。按党的政策纪律要求，加强政治修养，规范个人行为，严于律己，严格约束家人和身边工作人员，树立自身过硬、风清气正的优良作风，提高领导干部的凝聚力、感召力、公信力。要强化工作落实，真抓实干，真正不辱使命、不负重托，把政府各项工作做好，保障经济社会又好又快、更好更快发展和各方面工作全面进步升级取得更大成效。

　　各位代表、各位同志，乘党的十七大开启中国特色社会主义事业新的伟大征程的强劲东风，今天的鹤岗是东风吹来满城春，机遇前所未有，希望前所未有，困难和风险也前所未有。让我们高举中国特色社会主义伟大旗帜，坚持以邓小平理论、“三个代表”重要思想为指导，全面贯彻落实科学发展观，在省委、省政府和市委的正确领导下，依靠集体的力量、全市人民的智慧，以更加开放、更加广阔的胸怀去争取迎接更大领域、更多更大的支持，营造更多更强的优势，抢抓机遇、乘势而上、众志成城、合力强力攻坚，全力推动经济社会又好又快、更好更快发展、各方面工作更大幅度的全面进步升级，为城市创新转型、人民富裕幸福、建设边疆名城做出新的更大的贡献！
